# Supplementary material for: The burden of premature mortality from cardiovascular diseases: A systematic review of years of life lost
Source: PLoS One. 2023 Apr 21;18(4):e0283879. doi: 10.1371/journal.pone.0283879 (PMC10121009; doi:10.1371/journal.pone.0283879)
Supplement: S1 Table — (DOCX) [file pone.0283879.s003.docx]

**Supplement 2**

**Table: Search Terms**

| Databases | Search terms |
| --- | --- |
| Web of Science | AB=("cardiovascular disease*" OR "CVD" OR "coronary disease*" OR "coronary heart disease*" OR "heart disease*" OR "cardiac disease*" OR "cardiac disorder*" OR "heart disorder*" OR "cardiac arrhythmia*" OR "cardiac dysrhythmia*" OR "atrial fibrillation*" OR "coronary artery disease*" OR "coronary arteriosclerosis" OR "Coronary Atherosclerosis" OR "myocardial ischemia*" OR "ischemic heart disease*" OR "myocardial infarction*" OR "cardiovascular stroke*" OR "heart attack*" OR "cardiogenic shock" OR "acute coronary syndrome*" OR "angina pectori*" OR "Cerebrovascular Disorders" OR "Cerebrovascular Disorders" OR "intracranial vascular disease*" OR "intracranial vascular disorder*" OR "cerebrovascular disease*" OR "brain vascular disorder*" OR "cerebrovascular occlusion*" OR "cerebrovascular insufficiency*" OR "brain ischemia" OR "carotid artery disease*" OR "cerebral vessel diseases" OR "intracranial arterial diseases" OR "intracranial hemorrhage*"OR "cerebral hemorrhage*" OR "stroke*" OR "cerebrovascular accident*" OR "CVA" OR "brain vascular accident*" OR "intracranial arteriosclerosis" OR "cerebral arteriosclerosis" OR "cerebral atherosclerosis" )  AND  TI=("premature mortality" OR "premature death" OR "years of life lost" OR "YLL" OR "Potential Years of Life Lost" OR "PYLL" OR "life expectancy " )  AND  Articles (Document Types) and English (Languages) and Review Articles (Exclude – Document Types) and Animals or Mice (Exclude – MeSH Headings) |
| Pubmed | Search: cardiovascular disease[MeSH Terms] OR "cardiovascular disease*"[Title/Abstract] OR "CVD"[Title/Abstract] OR "coronary disease*"[Title/Abstract] OR "coronary heart disease*"[Title/Abstract] OR "heart disease*"[Title/Abstract] OR "cardiac disease*"[Title/Abstract] OR "cardiac disorder*"[Title/Abstract] OR "heart disorder*"[Title/Abstract] OR "cardiac arrhythmia*"[Title/Abstract] OR "cardiac dysrhythmia*"[Title/Abstract] OR "atrial fibrillation*"[Title/Abstract] OR "coronary artery disease*"[Title/Abstract] OR "coronary arteriosclerosis"[Title/Abstract] OR "Coronary Atherosclerosis"[Title/Abstract] OR "myocardial ischemia*"[Title/Abstract] OR "ischemic heart disease*"[Title/Abstract] OR "myocardial infarction*"[Title/Abstract] OR "cardiovascular stroke*"[Title/Abstract] OR "heart attack*"[Title/Abstract] OR "cardiogenic shock"[Title/Abstract] OR "acute coronary syndrome*"[Title/Abstract] OR "angina pectori*"[Title/Abstract] OR "Cerebrovascular Disorders"[Title/Abstract] OR "intracranial vascular disease*"[Title/Abstract] OR "intracranial vascular disorder*"[Title/Abstract] OR "cerebrovascular disease*"[Title/Abstract] OR "brain vascular disorder*"[Title/Abstract] OR "cerebrovascular occlusion*"[Title/Abstract] OR "cerebrovascular insufficiency*"[Title/Abstract] OR "brain ischemia"[Title/Abstract] OR "carotid artery disease*"[Title/Abstract] OR "cerebral vessel diseases"[Title/Abstract] OR "intracranial arterial diseases"[Title/Abstract] OR "intracranial hemorrhage*"OR "cerebral hemorrhage*"[Title/Abstract] OR "stroke*"[Title/Abstract] OR "cerebrovascular accident*"[Title/Abstract] OR "CVA"[Title/Abstract] OR "brain vascular accident*"[Title/Abstract] OR "intracranial arteriosclerosis"[Title/Abstract] OR "cerebral arteriosclerosis"[Title/Abstract] OR "cerebral atherosclerosis"[Title/Abstract]  AND  Search: "premature mortality"[Title/Abstract] OR "premature death"[Title/Abstract] OR "years of life lost"[Title/Abstract] OR "YLL"[Title/Abstract] OR "Potential Years of Life Lost"[Title/Abstract] OR "PYLL"[Title/Abstract] OR "life expectancy loss" [Title/Abstract] OR "standardized mortality ratios"[Title/Abstract] OR "standardized mortality rate*"[Title/Abstract] OR "Life Expectancy"[Title/Abstract]  AND  Limit to English and limit to Human |
| Scopus | TITLE-ABS-KEY ( "cardiovascular disease*" OR "CVD" OR "coronary disease*" OR "coronary heart disease*" OR "heart disease*" OR "cardiac disease*" OR "cardiac disorder*" OR "heart disorder*" OR "cardiac arrhythmia*" OR "cardiac dysrhythmia*" OR "atrial fibrillation*" OR "coronary artery disease*" OR "coronary arteriosclerosis" OR "Coronary Atherosclerosis" OR "myocardial ischemia*" OR "ischemic heart disease*" OR "myocardial infarction*" OR "cardiovascular stroke*" OR "heart attack*" OR "cardiogenic shock" OR "acute coronary syndrome*" OR "angina pectori*" OR "Cerebrovascular Disorders" OR "Cerebrovascular Disorders" OR "intracranial vascular disease*" OR "intracranial vascular disorder*" OR "cerebrovascular disease*" OR "brain vascular disorder*" OR "cerebrovascular occlusion*" OR "cerebrovascular insufficiency*" OR "brain ischemia" OR "carotid artery disease*" OR "cerebral vessel diseases" OR "intracranial arterial diseases" OR "intracranial hemorrhage*" OR "cerebral hemorrhage*" OR "stroke*" OR "cerebrovascular accident*" OR "CVA" OR "brain vascular accident*" OR "intracranial arteriosclerosis" OR "cerebral arteriosclerosis" OR "cerebral atherosclerosis" )  AND  TITLE ( "premature mortality" OR "premature death" OR "years of life lost" OR "YLL" OR "Potential Years of Life Lost" OR "PYLL" OR "life expectancy loss" )  AND  ( LIMIT-TO ( DOCTYPE , "ar" ) ) AND ( LIMIT-TO ( LANGUAGE , "english" ) ) |
| Cochrane Central Register of Controlled Trials (CENTRAL) | MeSH descriptor: [Cardiovascular Diseases] explode all trees  AND  "premature mortality" OR "premature death" OR "years of life lost" OR "YLL" OR "Potential Years of Life Lost" OR "PYLL" OR "life expectancy loss" |
