# Supplementary material for: The burden of premature mortality from cardiovascular diseases: A systematic review of years of life lost
Source: PLoS One. 2023 Apr 21;18(4):e0283879. doi: 10.1371/journal.pone.0283879 (PMC10121009; doi:10.1371/journal.pone.0283879)
Supplement: S3 Table — (DOCX) [file pone.0283879.s005.docx]

**Supplement 4**

The original version of the Newcastle-Ottawa Scale (NOS) was based on a cohort study and a case-control study design [1]. We used the adapted NOS version by Herzog et al. [2] for the cross-sectional study design, where they assess the same three components (selection, comparability, and outcome) as the original version. The table below presents both the original version and the adapted NOS version for a cross-sectional study.

**Table: Newcastle-Ottawa Scale for quality assessment of selected studies**

| Components | Newcastle-Ottawa Quality Assessment Scale original version [1] | Newcastle-Ottawa Scale adapted for cross-sectional studies [2] |
| --- | --- | --- |
| Selection | 1) Representativeness of the exposed cohort  a) truly representative of the average (describe) in the community *  b) somewhat representative of the average in the community *  c) selected group of users eg nurses, volunteers  d) no description of the derivation of the cohort  2) Selection of the non-exposed cohort  a) drawn from the same community as the exposed cohort *  b) drawn from a different source  c) no description of the derivation of the non-exposed cohort  3) Ascertainment of exposure  a) secure record (eg surgical records) *  b) structured interview *  c) written self-report  d) no description  4) Demonstration that outcome of interest was not present at start of study  a) yes *  b) no | 1) Representativeness of the sample:  a) Truly representative of the average in the target population. *  (all subjects or random sampling)  b) Somewhat representative of the average in the target population. *  (non-random sampling)  c) Selected group of users.  d) No description of the sampling strategy.  2) Sample size:  a) Justified and satisfactory. *  b) Not justified.  3) Non-respondents:  a) Comparability between respondents and non-respondents’ characteristics is established, and the response rate is satisfactory. *  b) The response rate is unsatisfactory, or the comparability between respondents and non-respondents is unsatisfactory.  c) No description of the response rate or the characteristics of the responders and the non-responders.  4) Ascertainment of the exposure (risk factor):  a) Validated measurement tool. **  b) Non-validated measurement tool, but the tool is available or described.*  c) No description of the measurement tool.  (Maximum 5 stars) |
| Comparability | 1) Comparability of cohorts on the basis of the design or analysis  a) study controls for (select the most important factor) *  b) study controls for any additional factor * (This criteria could be modified to indicate specific control for a second important factor.) | 1) The subjects in different outcome groups are comparable, based on the study design or analysis. Confounding factors are controlled.  a) The study controls for the most important factor (select one). *  b) The study control for any additional factor. *  (Maximum 2 stars) |
| Outcome | 1) Assessment of outcome  a) independent blind assessment *  b) record linkage *  c) self-report  d) no description  2) Was follow-up long enough for outcomes to occur  a) yes (select an adequate follow up period for outcome of interest) *  b) no  3) Adequacy of follow up of cohorts  a) complete follow up - all subjects accounted for *  b) subjects lost to follow up unlikely to introduce bias - small number lost - > ____ % (select an adequate %) follow up, or description provided of those lost) *  c) follow up rate < ____% (select an adequate %) and no description of those lost | 1) Assessment of the outcome:  a) Independent blind assessment. **  b) Record linkage. **  c) Self report. *  d) No description.  2) Statistical test:  a) The statistical test used to analyze the data is clearly described and appropriate, and the measurement of the association is presented, including confidence intervals and the probability level (p value). *  b) The statistical test is not appropriate, not described or incomplete.  (Maximum 3 stars) |

References

1. Wells GA, Shea B, O’Connell D, Peterson J, Welch V, Losos M, et al. The Newcastle-Ottawa Scale (NOS) for assessing the quality of nonrandomised studies in meta-analyses. Oxford; 2000.
2. Herzog R, Álvarez-Pasquin Maand D\’\iaz C, del Barrio JL, Estrada JM, Gil Á. Are healthcare workers’ intentions to vaccinate related to their knowledge, beliefs and attitudes? A systematic review. BMC Public Health. 2013;13: 1–17.
